# In situ structural analysis of SARS-CoV-2 spike reveals flexibility mediated by three hinges

**DOI:** 10.1126/science.abd5223

**Published:** 2020-08-18

**Authors:** Beata Turoňová, Mateusz Sikora, Christoph Schürmann, Wim J. H. Hagen, Sonja Welsch, Florian E. C. Blanc, Sören von Bülow, Michael Gecht, Katrin Bagola, Cindy Hörner, Ger van Zandbergen, Jonathan Landry, Nayara Trevisan Doimo de Azevedo, Shyamal Mosalaganti, Andre Schwarz, Roberto Covino, Michael D. Mühlebach, Gerhard Hummer, Jacomine Krijnse Locker, Martin Beck

**Affiliations:** 1 Structural and Computational Biology Unit, European Molecular Biology Laboratory (EMBL), Meyerhofstr. 1, 69117 Heidelberg, Germany.; 2 Department of Molecular Sociology, Max Planck Institute of Biophysics, Max-von-Laue Str. 3, 60438 Frankfurt am Main, Germany.; 3 Department of Theoretical Biophysics, Max Planck Institute of Biophysics, Max-von-Laue Str. 3, 60438 Frankfurt am Main, Germany.; 4 Division of Veterinary Medicine, Paul Ehrlich Institute, Paul Ehrlich Strasse 51-59, 63225 Langen, Germany.; 5 Central Electron Microscopy Facility, Max Planck Institute of Biophysics, Max-von-Laue Str. 3, 60438 Frankfurt am Main, Germany.; 6 Division of Immunology, Paul Ehrlich Institute, Paul Ehrlich Strasse 51-59, 63225 Langen, Germany.; 7 German Center for Infection Research, Gießen-Marburg-Langen, Germany.; 8 Institute for Immunology, University Medical Center, Johannes Gutenberg University Mainz, Mainz, Germany.; 9 Research Center for Immunotherapy (FZI), University Medical Center, Johannes Gutenberg-University Mainz, Mainz, Germany.; 10 Genomics Core Facility, EMBL, Meyerhofstr. 1, 69117 Heidelberg, Germany.; 11 Frankfurt Institute for Advanced Studies, Ruth-Moufang-Str. 1, 60438 Frankfurt am Main, Germany.; 12 Institute of Biophysics, Goethe University Frankfurt, 60438 Frankfurt am Main, Germany.; 13 Electron Microscopy of Pathogens Unit, Paul Ehrlich Institute, Paul Ehrlich Strasse 51-59, 63225 Langen, Germany.

## Abstract

The spike protein (S) of severe acute respiratory syndrome coronavirus 2 (SARS-CoV-2) is required for cell entry and is the primary focus for vaccine development. In this study, we combined cryo–electron tomography, subtomogram averaging, and molecular dynamics simulations to structurally analyze S in situ. Compared with the recombinant S, the viral S was more heavily glycosylated and occurred mostly in the closed prefusion conformation. We show that the stalk domain of S contains three hinges, giving the head unexpected orientational freedom. We propose that the hinges allow S to scan the host cell surface, shielded from antibodies by an extensive glycan coat. The structure of native S contributes to our understanding of SARS-CoV-2 infection and potentially to the development of safe vaccines.

## Flexible spikes

The severe acute respiratory syndrome coronavirus 2 (SARS-CoV-2) spike protein enables viral entry into host cells by binding to the angiotensin-converting enzyme 2 (ACE2) receptor and is a major target for neutralizing antibodies. About 20 to 40 spikes decorate the surface of virions. Turoňová *et al.* now show that the spike is flexibly connected to the viral surface by three hinges that are well protected by glycosylation sites. The flexibility imparted by these hinges may explain how multiple spikes act in concert to engage onto the flat surface of a host cell.

*Science*, this issue p. 203

## Teaser

Flexible hinges shielded by glycans in the coronavirus spike protein may allow scanning of the host cell surface.

The spike surface protein (S) of the severe acute respiratory syndrome coronavirus 2 (SARS-CoV-2) is required to initiate infection (*1*). It binds to the angiotensin-converting enzyme 2 (ACE2) (*2*, *3*) to mediate viral entry. S also determines tissue and cell tropism. Mutations may alter the host range of the virus and enable the virus to cross species barriers (*4*, *5*). Vaccine efforts focus on neutralizing antibodies that block infection by binding to S.

S is a trimeric class I viral fusion protein (*6*) with a club-like shape of ~20 nm in length. The ectodomain consists of a head, which has been extensively studied in vitro. It is connected to the membrane by a slender stalk. The three receptor binding domains (RBDs) of the S head are conformationally variable, which may relate to receptor binding. In the closed conformation, the RBDs are shielded by the N-terminal domains (NTDs). In the open conformation, one RBD is exposed upward away from the viral membrane (*2*, *3*)*.* Previous studies resolved roughly two-thirds of the predicted 22 N-linked glycans that are thought to shield S against antibodies (*2*, *3*). It remains unknown whether the distribution of the conformational states and the glycosylation pattern observed with recombinant protein in vitro are representative of the native state generated during viral assembly. Furthermore, little is known about the stalk of S and how its conformational variability within the virion may affect the accessibility of epitopes for neutralizing antibodies and facilitate viral entry.

## SARS-CoV-2 virions present prefusion S in an irregular pattern

**Fig. 1:**
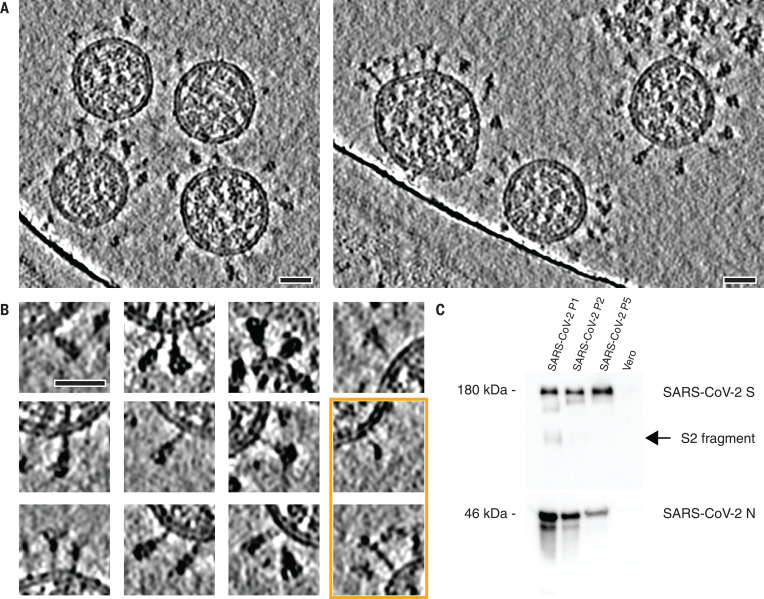
Cryo–electron tomography of SARS-CoV-2 virions. (**A**) Slices through tomographic reconstructions of SARS-CoV-2 virions. Scale bars, 30 nm. (**B**) Same as (A), but tomograms are arranged as a gallery to highlight specific features of S. All domains, including the transmembrane part, are clearly resolved. Although most of S is reminiscent of the prefusion conformation, the particles framed in orange resemble the postfusion conformation as described by Cai *et al*. (*7*). Scale bar, 30 nm. (**C**) Immunoblot showing the loss of cleavage products of SARS-CoV-2 S with uncleaved S (180 kDa) remaining, within five passages (P1 to P5) through tissue culture (loading control using anti-N antibody). N, nucleocapsid protein.

**Fig. 2:**
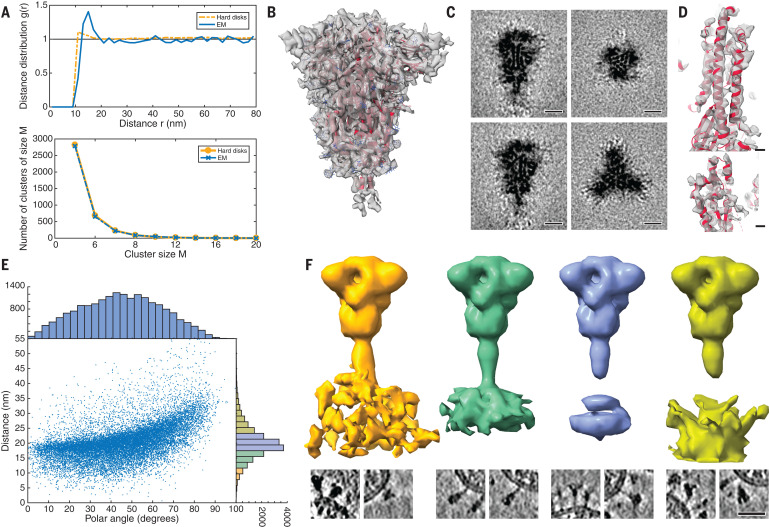
Subtomogram analysis of SARS-CoV-2 S protein. (**A**) Distance (top) and cluster-size distributions (bottom) of S on the viral surface, with nonoverlapping hard disks of 10-nm diameter as a reference. (**B**) Subtomogram average of the ectodomain of S, shown isosurface rendered and fitted with the previously published atomic model as determined by single-particle EM (PDB ID 6VXX). Transparent gray, subtomogram average; red, secondary structure elements; blue, glycosylation sites. (**C**) Same subtomogram average but shown as slices through the reconstruction. Scale bars, 5 nm. (**D**) Detail of the average of the symmetric unit of S. Scale bars, 5 Å. (**E**) Distribution of the angular orientation and distance of the ectodomain with respect to the bilayer. (**F**) On the basis of the initial subtomogram averaging, positions of the spike head were classified according to their distance from the lipid bilayer (supplementary materials). (Top) Averages of the resulting classes are shown isosurface rendered; distance increases from left to right. (Bottom) Examples of individual particles are shown as slices. At an optimal distance, the stalk domain stretches out and is resolved. Scale bar, 30 nm.

To structurally analyze SARS-CoV-2 S in situ, we passaged the virus through tissue culture cells and used sucrose centrifugation to purify it from the inactivated supernatant (see materials and methods). We acquired a large-scale cryo–electron tomography dataset that consists of 266 tilt series covering >1000 viruses. Visual inspection of the tomographic reconstructions revealed a very-high-quality data set in which individual protein domains were clearly visible (Fig. 1A and movie S1). On average, 40 copies of the S trimer resided on the surface. S proteins appeared to be distributed randomly on the viral surface without any significant tendency to cluster (Fig. 2A).

S was mostly present in the prefusion conformation (Fig. 1B). Postfusion conformations (*7*, *8*) were very rare (<0.1%), which appears typical for Vero E6 host cells (*9*). Sanger sequencing and immunoblot analysis revealed that the furin site for proteolytic cleavage into the S1 and S2 fragments (*5*, *10*) was lost during tissue culture passage (Fig. 1C and fig. S1), consistent with previous studies (*11*, *12*)*.* However, the isolate contained the Asp^614^→Gly (D614G) allele (*13*, *14*)*.* Large-scale sequencing of RNA isolated from tissue culture supernatant confirmed both findings (supplementary materials).

Subtomogram averaging with NovaSTA (*15*) and STOPGAP (*16*) resulted in a cryo–electron microscopy (cryo-EM) map of the S head at 7.9 Å resolution (fig. S2), in which secondary structure elements and individual glycosylation sites were clearly discernible (Fig. 2, B and C). Classification suggested that about half of S was present in the fully closed conformation. A considerable fraction of the remaining subtomograms had one RBD exposed (fig. S3). Structural analysis of the asymmetric unit yielded an average map of the closed conformation at an overall resolution of 4.9 Å. In particular, the cluster of parallel helices in the center of the head was clearly resolved (Fig. 2D and fig. S4).

By contrast, the stalk connecting the S head to the viral membrane appeared to be dynamic. Although the head was fully contained in the tomographic map, only the top of the stalk domain was resolved. Emerging from the neck of the spike head, it contains an 11-residue Leu repeat sequence (L1141, L1145, and L1152) and adopts an unusual right-handed coiled coil, consistent with a recent single-particle structure of the S head (*7*)*.* We will henceforth refer to this part of the stalk domain as the “upper leg.” Right-handed trimeric coiled coils were long thought to be absent from the structural proteome (*17*) but can be seen in the postfusion structure of S from the related mouse hepatitis virus (*18*).

## A stalk with three flexible hinges connects S to the viral membrane

The tomographic images suggest the presence of flexible hinges in the stalk. Stalks of individual S proteins are clearly visible in the tomograms (Fig. 1B), but, after averaging, their density declined sharply at the end of the trimeric coiled coil that forms the upper leg (Fig. 2B). Moreover, the head exhibited large positional and orientational freedom. It was tilted up to ~90° with respect to the normal at distances of 5 to 35 nm from the membrane (Fig. 2E). We grouped our subtomograms into four classes, according to their distance from the bilayer, and averaged them separately. At an intermediate distance, parts of the stalk and bilayer were resolved, suggesting a more defined conformation (Fig. 2F). We then subselected ~3200 particles in which the head was oriented roughly perpendicular to the membrane. In the resulting average, the stalk domain was resolved (fig. S5A). Visual inspection of the respective subtomograms, in which the stalk domains are clearly observed, further corroborated the idea of a kinked stalk with potentially several hinges (Fig. 2F). Local refinement of the lower part of the stalk (henceforth referred to as the “lower leg”) resulted in a moderately resolved structure that would be consistent with the continuation of the coiled coil below a flexible hinge (henceforth referred to as the “knee”) (fig. S5B).

**Fig. 3:**
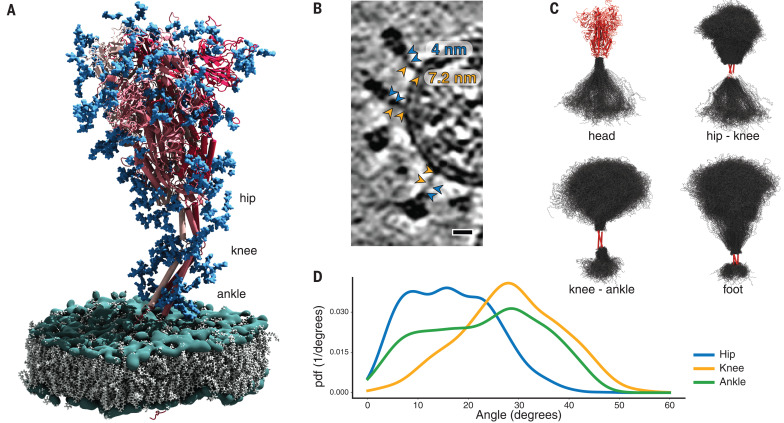
MD simulations of SARS-CoV-2 S protein. (**A**) Model of the S protein. The three individual chains of S are shown in shades of red, N-glycosylation in blue, lipids of the endoplasmic reticulum–like membrane in gray, and phosphates in green. “Hip,” “knee,” and “ankle” mark positions of the three flexible hinges. (**B**) Examples of the hinges as seen in the deconvoluted tomograms. Blue and orange arrowheads indicate the upper and lower legs, respectively, with their typical lengths indicated. Scale bar, 10 nm. (**C**) Hinge flexibility in the MD simulation illustrated through backbone traces (gray) at 75-ns intervals with different parts of the S protein fixed (red). (**D**) Probability density functions (pdf) for hinge bending angles at the hip, knee, and ankle.

Molecular dynamics (MD) simulations helped us to pinpoint the molecular origins of the flexibility seen in the tomograms. We performed a 2.5-μs-long all-atom MD simulation of a 4.1 million atom system containing four glycosylated S proteins anchored into a patch of viral membrane and embedded in aqueous solvent (Fig. 3A). In the simulations, the S heads remained stable. The stalks, however, exhibited pronounced hinging motions at the junctions between the S head and the upper leg (“hip”), between the upper and lower legs (“knee”), and between the lower leg and the transmembrane domain (“ankle”). This observation was consistent with discrete leg segments seen in the raw tomograms (Fig. 3, B and C). The hip joint flexed the least (16.5° ± 8.8°), followed by the ankle (23.0° ± 11.7°) and the knee (28.4° ± 10.2°) (Fig. 3D and fig. S6). However, the limited sampling in the MD simulation may not have covered the full range of motions (compare Fig. 2E and fig. S6D).

**Fig. 4:**
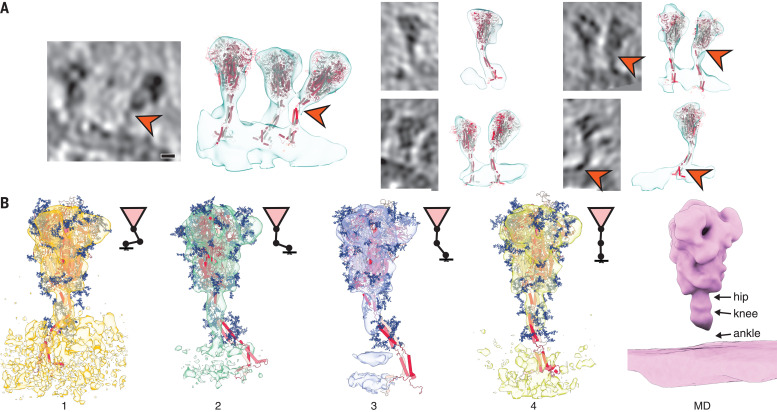
Fitting of molecular simulations into cryo–electron tomograms. (**A**) Slices through tomograms (left) and isosurface-rendered tomograms with snapshots of the respective MD simulations superimposed without flexible fitting (right). The hinges of the stalk domain predicted by structural modeling (orange arrowheads) are consistent with the tomographic data. Scale bar, 5 nm. (**B**) Fit of snapshots of MD simulations into the classes obtained for different distances of the head from the membrane (1 to 4), as presented in Fig. 2F. Shorter distances are concomitant with a stronger bending of the hinges and a lateral displacement of the stalk. Average MD density filtered to a resolution comparable to the subtomogram averages is shown as an isosurface rendering (right).

Structures of S seen along the MD trajectory fit well into the tomographic density of S proteins protruding from the viral surface (Fig. 4A). In particular, the joints of the hip, knee, and ankle of the MD snapshots aligned with kinks in the density visualized by cryo-EM. For a more detailed view, we flexibly fitted suitable snapshots of the MD simulations into the subtomogram averages classified according to the distance of the head from the membrane (compare Fig. 2F to Fig. 4B). Hinge bending gives the S stalk the flexibility required to connect heavily tilted S heads to the viral membrane.

As a result of hinge bending, the stalk is diluted out in subtomogram averages focused on the head (Figs. 2, B, C, and F, and 4B). Stalks were visible if the heads were aligned with the membrane normal (fig. S5A) or if the stalks themselves were averaged separately (fig. S5B). To test this interpretation, we calculated the electron density averaged over the entire MD trajectory with aligned S heads. Filtered to a comparable resolution, this calculated 3D map was highly similar to the subtomogram averages (Fig. 4B). In rare cases, the coiled coil near the membrane appears to be unfolded in the original tomograms (fig. S5C) and continuous with the disordered loops of the MD model.

## Extensive N-glycosylation covers the surface of S

**Fig. 5:**
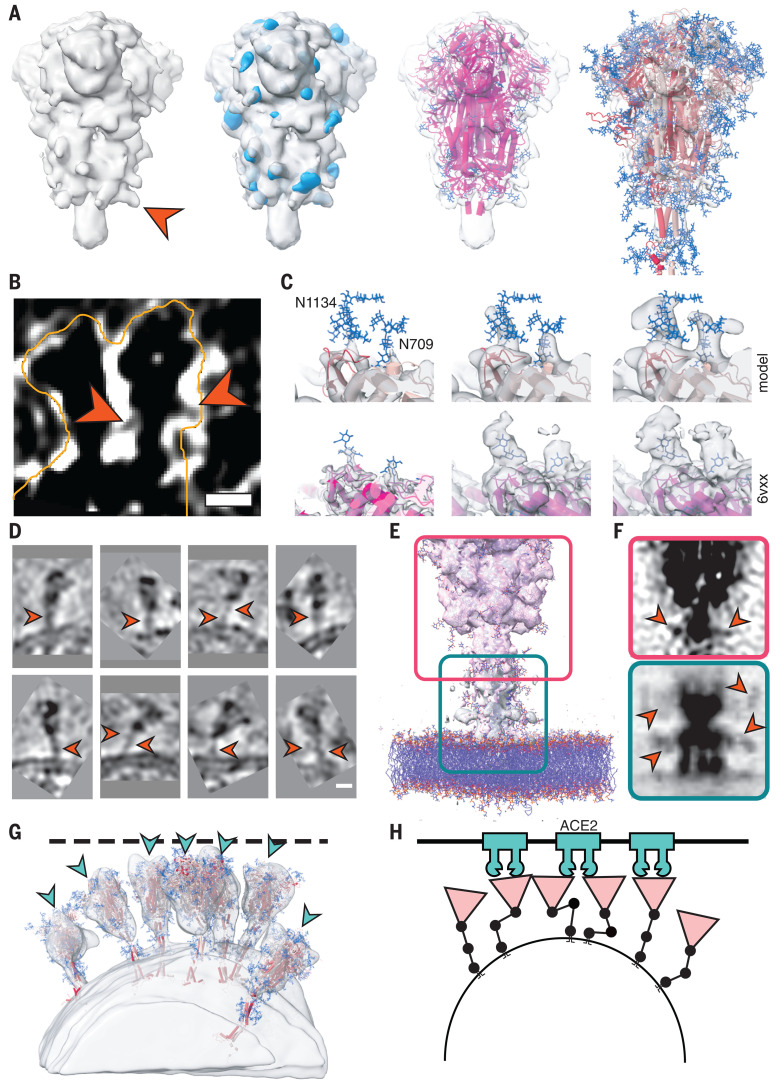
Analysis of S protein glycosylation sites and epitopes. (**A**) N-glycosylation sites are clearly discernible in the subtomogram average of the head. From left to right: Isosurface rendering of subtomogram average with an individual N-glycosylation site indicated (orange arrowhead); superimposed with the MD-calculated density for all annotated N-glycosylation sites; superimposed with previous structural model of the head (PDB ID 6VXX); and superimposed with a snapshot of the MD simulations. N-glycosylation sites are shown in blue. (**B**) Tomographic slice highlighting an N-glycosylation site (orange arrowheads) in the original data. Scale bar, 5 nm. (**C**) Highlight of N-glycosylation positions 709 and 1134 of the MD simulations (top) and in a previous structural model (bottom; PDB ID 6VXX, EMDB 21452). The subtomogram average is shown superimposed at different isosurface thresholds (transparent gray). Extensive additional density is visible. (**D** to **F**) The stalk domain is heavily glycosylated at the hinges. (D) Example tomographic slices with bulky density at the hinge positions (orange arrowheads). Scale bar, 5 nm. (E) Superposition of the subtomogram averages (transparent gray isosurfaces) of the head (framed red) and the stalk domain (framed green), with a respective snapshot of the MD simulations emphasizing the glycosylation at the hinges. (F) Same as (E) but shown as a maximum intensity projection through the subtomogram averages. Orange arrowheads indicate bulky density at hinges. (**G**) Fits of snapshots from MD simulations into the surface of a virion. The tomogram is shown isosurface rendered in transparent gray. The position of epitopes for neutralizing antibodies at the RBDs are indicated with blue arrowheads. (**H**) Cartoon illustrating a hypothetical docking event in which the hinges facilitate the engagement of multiple instances of S with their receptors.

The predicted N-glycosylation sites, many already annotated in single-particle EM maps (*2*), were generally very pronounced in the subtomogram averages. The electron density of N-glycans averaged over the MD trajectory was highly consistent with the tomographic map (Fig. 5A). Clustered glycosylation sites were visible in the raw density before averaging (e.g., protruding from the lower part of the S head; Fig. 5B). Our analysis of individual sites in subtomogram averages further supports the notion that the spikes were decorated with rather bulky glycan chains (Fig. 5C). Notably, a number of sequons were resolved with more-pronounced branching than previously reported (*19*). By contrast, the two predicted O-glycosylation sites (*20*) lacked excess density (fig. S7A). Sequon N17LT, owing to its location on the unstructured N terminus, was not localized in the density (fig. S7B), but elongated features protruding from the tip of the N-terminal domain (fig. S7B) suggested the presence of sequons N74GT and N149KS.

N-glycosylation is also predicted on the knee (N1158HT and N1173AS) and the ankle (N1194ES) in regions not previously resolved by single-particle EM (Fig. 3A). We observed that these positions generally appeared bulkier in tomographic reconstructions than one might expect if they were not glycosylated (Figs. 1B and 5D). Additional density was very clearly observed in subtomogram averages (Fig. 5, E and F, and fig. S5, A and B), and consistent electron density calculated from the MD trajectory aligned on the lower leg (fig. S7C). N-glycosylation in this region of S might protect the functionally important hinges from antibody binding and help to keep them flexible.

## Discussion

The two primary structural analysis techniques combined in this study are complementary. Our MD simulations revealed three flexible hinges (hip, knee, and ankle) within the stalk, consistent with the tomographic data. One might speculate that the high degree of conformational freedom of S on the viral surface is important for the mechanical robustness of the virus or may facilitate motions that interfere with antibody access to the stalk. It might also allow S to engage the relatively flat surface of host cells with higher avidity (Fig. 5, G and H). Future tomographic studies of actual infection events might further explore these topics. In contrast to the prefusion conformation of S, the postfusion conformation previously observed in vitro and in situ (*7*, *9*), as well as in this study (Fig. 1B), is apparently inflexible. To the best of our knowledge, extensive flexibility comparable to that of the prefusion S stalk has not been reported for other class I viral fusion proteins, including HIV env, influenza HA, or Ebola GP. However, influenza HA attaches to micelles with a short linker permitting up to 25° bending (*21*).

A particularly unusual feature masked at the edge of the resolved density of single-particle structures but well resolved in the subtomogram averages is the short right-handed coiled coil at the top of the prefusion stalk. Because this feature is lost in the postfusion structure as resolved for SARS-CoV (*8*), we speculate that it is only marginally stable, priming the protein for a large structural reorganization in a spring-loaded viral fusion mechanism. Indeed, all three hinges are disassembled in the transition to the postfusion conformation and placed outside the structural core (*7*, *8*)*.*

Overall, the observed distribution of S on the surface of the virion and its conformers is highly consistent with the findings of other studies (*9*, *22*, *23*). Host cell-type–dependent differences in the abundance of pre- and postfusion conformation (*9*, *22*) may depend on different levels of ACE2 and the serine protease TMPRSS2 (*10*). Whether the furin cleavage site plays a role here remains to be addressed. A notable difference is the higher abundance of S on the viral surface observed in this study compared with others (*22*, *23*).

The fully closed prefusion conformation of S was abundant in situ. This finding emphasizes that the highly engineered, recombinant versions of S locked into this conformation (*24*, *25*) may be valuable tools for vaccine development, although there are also differences to the in situ structure. N-glycosylation sites appeared very bulky in the tomographic map compared with previous single-particle analysis, suggesting that decoration with sugars may be more extensive on S assembled in infected cells than on S expressed recombinantly. Our map is suggestive of additional N-glycosylation at the hinges of the stalk domain and possibly on the tips of the S NTDs. The native glycosylation pattern defines the accessibility of epitopes on the crowded viral surface (*19*), where the NTD and stalk domains appear occluded by neighboring spikes (Fig. 5G). A lack of excess density at the predicted O-glycosylation sites indicates that N-glycosylation dominates.

By using cryo–electron tomography of intact viruses, we were able to resolve functionally important parts of S, including its connection to the viral membrane and its glycan coat, which were masked in studies of recombinant detergent-solubilized protein. Beyond S, our large-scale tomographic dataset contains rich, high-resolution structural information on SARS-CoV-2 particles in their native context. The in situ structures of several key viral components—including the nucleocapsid and the M protein that is highly enriched in the membrane—remain enigmatic. Our data might thus be explored to resolve such features in the future. Furthermore, high-resolution structural models can be fitted directly into the tomographic reconstructions, emphasizing the high quality of the data. This strategy might thus help us to build structural models of entire virions.

## Supplementary Materials

science.sciencemag.org/content/370/6513/203/suppl/DC1 Materials and Methods Figs. S1 to S7 Tables S1 and S2 References (*26*–*60*) MDAR Reproducibility Checklist Movie S1

## Appendix

View/request a protocol for this paper from *Bio-protocol*.
